# Lineage‐specific mechanisms and drivers of breast cancer chemoresistance revealed by 3D biomimetic culture

**DOI:** 10.1002/1878-0261.13037

**Published:** 2021-07-10

**Authors:** Chiara Liverani, Alessandro De Vita, Chiara Spadazzi, Giacomo Miserocchi, Claudia Cocchi, Alberto Bongiovanni, Anna De Lucia, Federico La Manna, Francesco Fabbri, Michela Tebaldi, Dino Amadori, Ennio Tasciotti, Giovanni Martinelli, Laura Mercatali, Toni Ibrahim

**Affiliations:** ^1^ Osteoncology and Rare Tumors Center Istituto Scientifico Romagnolo per lo Studio e la Cura dei Tumori (IRST) IRCCS Meldola Italy; ^2^ Bioscience Laboratory Istituto Scientifico Romagnolo per lo Studio e la Cura dei Tumori (IRST) IRCCS Meldola Italy; ^3^ Unit of Biostatistics and Clinical Trials Istituto Scientifico Romagnolo per lo Studio e la Cura dei Tumori (IRST) IRCCS Meldola Italy; ^4^ Center for Biomimetic Medicine Houston Methodist Research Institute (HMRI) TX USA; ^5^ IRCCS San Raffaele Pisana Rome Sclavo Research Center Siena Italy; ^6^ Scientific Directory Istituto Scientifico Romagnolo per lo Studio e la Cura dei Tumori (IRST) IRCCS Meldola Italy

**Keywords:** 3D models, breast cancer, DNA repair, doxorubicin, drug resistance, lysosomes

## Abstract

To improve the success rate of current preclinical drug trials, there is a growing need for more complex and relevant models that can help predict clinical resistance to anticancer agents. Here, we present a three‐dimensional (3D) technology, based on biomimetic collagen scaffolds, that enables the modeling of the tumor hypoxic state and the prediction of *in vivo* chemotherapy responses in terms of efficacy, molecular alterations, and emergence of resistance mechanisms. The human breast cancer cell lines MDA‐MB‐231 (triple negative) and MCF‐7 (luminal A) were treated with scaling doses of doxorubicin in monolayer cultures, 3D collagen scaffolds, or orthotopically transplanted murine models. Lineage‐specific resistance mechanisms were revealed by the 3D tumor model. Reduced drug uptake, increased drug efflux, and drug lysosomal confinement were observed in triple‐negative MDA‐MB‐231 cells. In luminal A MCF‐7 cells, the selection of a drug‐resistant subline from parental cells with deregulation of p53 pathways occurred. These cells were demonstrated to be insensitive to DNA damage. Transcriptome analysis was carried out to identify differentially expressed genes (DEGs) in treated cells. DEG evaluation in breast cancer patients demonstrated their potential role as predictive biomarkers. High expression of the transporter associated with antigen processing 1 (*TAP1*) and the tumor protein p53‐inducible protein 3 (*TP53I3*) was associated with shorter relapse in patients affected by ER^+^ breast tumor. Likewise, the same clinical outcome was associated with high expression of the lysosomal‐associated membrane protein 1 *LAMP1* in triple‐negative breast cancer. Hypoxia inhibition by resveratrol treatment was found to partially re‐sensitize cells to doxorubicin treatment. Our model might improve preclinical *in vitro* analysis for the translation of anticancer compounds as it provides: (a) more accurate data on drug efficacy and (b) enhanced understanding of resistance mechanisms and molecular drivers.

## Graphical abstract

We present a 3D collagen‐based model used to predict *in vivo* chemotherapy responses and to reveal lineage‐specific drug resistance mechanisms in breast cancer. After doxorubicin treatment, reduced drug uptake and lysosomal confinement were observed in triple‐negative MDA‐MB‐231 cells whereas a subclone selection with deregulation of p53 pathways and reduced DNA damage response was shown in luminal A MCF‐7 cells.

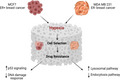

**Abbreviations**
ABC: ATP‐binding cassette
Ct: threshold cycle
DEGs: differentially expressed genes
ECMs: extracellular matrices
mTORC1: mammalian target of rapamycin complex 1
SEM: scanning electron microscopy

## 1 Introduction

Drug resistance is the major cause of treatment failure in cancer patients, ultimately leading to the death of patients with advanced‐stage cancers [1]. For decades, candidate drugs for clinical development have been tested on monolayer cultures using inert plastic supports. While this approach has benefit from a hit identification perspective, it presents some limitations: the fast‐replicating phenotype of cancer cells in this model enhances their sensitivity to antiproliferative drugs producing overestimated responses, while complex stimuli from the tumor microenvironment that are involved in multidrug resistance are lacking [2]. The association of a defective DNA repair machinery and exposure to oxidative stress affects cell genetic and epigenetic characteristics, altering key biological functions like cell metabolism and resistance profile [3]. This at least partially explains why the 90% of promising drug candidates that proved effective and safe in preclinical trials ultimately fail in the clinical setting [4]. To improve the success rate of current trials, efforts have been directed on two main areas: advances in precision medicine to introduce predictive biomarkers for the selection of patients that would benefit most from specific targeted therapies, and the development of engineered models to enhance the predictivity of preclinical tests through the recapitulation of key tumor features involved in therapy response [5]. In this regard, a plethora of three‐dimensional (3D) culture systems mimicking physical, chemical, and biological elements of the tumor microenvironment have been developed up to date [6, 7, 8, 9, 10]. These models allow to recreate and dissect: (a) the penetration and distribution of drugs in 3D structures [11, 12, 13, 14]; (b) how mechanical stimuli as the flow of extracellular fluids that generate flux of compounds and removal of metabolites impact drug efficacy [15]; (c) drug delivery strategies mediated by the targeting of extracellular matrices (ECMs) [16]; (d) the induction of resistance mechanisms by the tumor microenvironment and tumor stroma [17, 18] including immune cells [19, 20]; and (e) the preservation in native environments of primary cell phenotypes for precision medicine [21, 22]. In particular, it has been extensively demonstrated that culturing cancer cells in these engineered systems generates an enhanced understanding of resistance mechanisms that are generally difficult to study in monolayer conditions [23, 24, 25, 26, 27].

Drug resistance might be derived from intrinsic cancer cell characteristics or emerge during the tumor evolution driven by a number of different mechanisms, such as drug efflux or inactivation, target alteration, inhibition of cell death, or epithelial to mesenchymal transitions [28]. The subpopulation of drug‐resistant cells is often responsible for cancer relapse that follows the remission period after treatment. These cells persist in patients and might migrate to distant sites initiating metastasis [29]. Therefore, it is fundamental that drug development and screening processes have evolved from monolayer culture systems toward more complex and relevant models that can help predict clinical drug resistance [30]. The combination of these innovative experimental approaches with new generation genomic and proteomic technologies will help identify novel resistance mechanisms and study therapies that can overcome this process and target cells that are not susceptible to current treatments [31].

Breast cancer is one of the most frequent cancers and common causes of cancer‐related deaths among women [32]. Despite the advances in breast cancer treatment in early and metastatic phase, medical therapies still fail in patients due to pharmacological resistance, resulting in disease progression, recurrence, and reduced overall survival [33].

We previously established a 3D technology based on biomimetic scaffolds that mimic the hierarchically organized structure of extracellular collagen, a matrix protein that is present in almost every tissue of the body [34]. Biocompatible materials with high degree of ECM biochemical mimicking have been successfully used in different tissue engineering applications [35], as native ECMs provide fundamental stimuli affecting cell function during pathophysiological events, including cancer development and evolution [36, 37, 38, 39]. Our scaffolds enabled the modeling of the tumor hypoxic niche and its contribution to disease progression. We implemented this platform for the identification of lineage‐specific drug resistance mechanisms. Here, we have applied this approach in two established breast cancer models and identified mechanisms not yet fully described in literature. Clinically relevant biomarkers were investigated and generated to predict doxorubicin efficacy in patients.

## 2 Materials and methods

### 2.1 Collagen scaffold synthesis

All chemicals were purchased from Sigma‐Aldrich (St. Louis, MO, USA). The collagen scaffolds were synthesized and characterized as previously described [34]. Type I collagen was suspended in acetic acid, precipitated to pH 5.5, and cross‐linked with 1,4‐butanediol diglycidyl ether. An established freezing and heating ramp (from 25 °C to −25 °C and from −25 °C to 25 °C in 50 min under vacuum conditions, *P* = 0.20 mbar) produced the scaffold's porosity ensuring proper pore size, interconnectivity, and orientation. Scaffolds were sterilized in 70% ethanol for 1 h and then washed three times in sterile Dulbecco's Phosphate‐Buffered Saline (Life Technologies, Carlsbad, CA, USA). Porosity and pore size of the scaffold were determined as previously described [34].

### 2.2 Cell seeding and culture

The human breast cancer cell lines MDA‐MB‐231 and MCF‐7 were obtained from the American Type Culture Collection (Rockville, MD, USA). Cells were maintained in DMEM medium with 10% fetal bovine serum, 1% penicillin‐streptomycin, and 1% glutamine (PAA, Piscataway, NJ, USA) at 37 °C in a 5% CO_2_ atmosphere. For monolayer cultures, 6 × 10^5^ cells were seeded in 25‐cm^2^ flasks. For 3D cultures, 5 × 10^6^ cells suspended in 50 µL of culture medium were dropped onto the upper surface of each scaffold (1 × 9 mm) in 24 multiwell plates. Scaffolds were plunged in PBS to maintain them hydrated. Before cell seeding, scaffolds were dried out through the elimination of liquid using sterile tips. Seeding was reached by soaking of the cell suspension in dry scaffolds. After cells were allowed to adhere for 1 h at 37 °C, the culture medium was gently added in each well. After 24 h, scaffolds were gently placed in a six multiwell plate. The medium was replaced daily. For the *in vivo* study, luciferase‐transfected MDA‐MB‐231 and MCF‐7 cells were maintained in DMEM high glucose without sodium pyruvate with 10% FCII (Fetal Clone II; Hyclone, Logan, UT, USA), 1% penicillin/streptomycin, 1% glutamine, and 800 µg·mL^−1^ of Geneticin (G418 Invitrogen, Waltham, MA, USA) for selection of luciferase and cultured as previously described [40].

### 2.3 Scanning electron microscopy and confocal microscopy

Cells in collagen scaffolds were imaged by scanning electron microscopy (SEM) and laser confocal microscopy as previously described [34]. For SEM, the samples were washed in 0.1 m sodium cacodylate buffer pH 7.4 and fixed in 2.5% glutaraldehyde 0.1 m sodium cacodylate buffer for 2 h at 4 °C. Before imaging, samples were dehydrated in a series of ethanol, dried in a dessicator overnight, and sputter‐coated with platinum. The Nova NanoSEM 230 (FEI, Hillsboro, OR, USA) was used to acquire all the images. For confocal microscopy cells were fixed in 4% paraformaldehyde for 20 min and stained with Alexa Fluor™ 546 Phalloidin and DAPI (Thermo Fisher Scientific, Waltham, MA USA) for 1 h at 4 °C. For lysosomes detection, cells were collected by trypsinization for monolayer cultures or by digestion in Collagenase type I (Merck Millipore, Burlington, MA, USA) for 3D culture. Cells were then stained with 75 nm LysoTracker™ Green DND‐26 (Thermo Fisher Scientific) for 30 min at 37 °C and cytospinned onto glass slides. For yH2AX immunofluorescence staining, Phospho‐Histone H2A.X (Ser139) (mAb #9718 Cell Signaling, Beverly, MA, USA) was used (1 : 400) and detected with secondary antibody Alexa Fluor™ 488. Images were acquired with an N‐SIM E laser confocal microscope (Nikon Corporation, Tokyo, Japan) and performed at 20× magnification.

### 2.4 Transcriptome analysis

The expression profile of 3D‐cultured MDA‐MB‐231 and MCF‐7 cells treated for 72 h with 4 µg·mL^−1^ doxorubicin was compared with that of untreated cells. Gene expression analysis was carried out using the Illumina HumanHT‐12 v4 Expression BeadChip (Illumina, San Diego, CA, USA). For RNA extraction, the scaffolds were fragmented into small pieces, while monolayer cultured cells were collected by tripsinization. RNA quality control was performed through an electrophoretic run on Agilent Bioanalyzer using the Agilent RNA 6000 Nano Kit (Agilent Technologies, Santa Clara, CA, USA). Total RNA samples were processed using the Ambion Illumina TotalPrep RNA Amplification kit. Beadchips were hybridized and processed following the Illumina Whole Genome Gene Expression Direct Hybridization Assay protocol. Fluorescence data generated by the iScan were analyzed with the Ilumina genomestudio software package. Data normalization was performed using the Robust Spline Normalization (RSN) algorithm. The identification of differentially expressed genes (DEGs) was addressed using Linear Models for Microarray Data (LIMMA) and empirical Bayes methods together with false discovery rate correction of the *P*‐value (Benjamini–Hochberg). Statistically significant DEGs (*P*.adj.value < 0.01) have been selected according to a |LogFC| > 1. We used KEGG (Kyoto Encyclopedia of Genes and Genomes), REACTOME, and Gene Ontology (GO) tools to test for the enrichment of any pathway/terms that may be related to the drug resistance phenotypes. For each tool, we have taken into consideration the first twenty terms sorted by adjusted *P*‐value.

### 2.5 Quantitative real‐time reverse transcriptional‐PCR (qRT‐PCR)

Total mRNA was isolated using TRIzol Reagent (Invitrogen) following the manufacturer's instructions and reverse‐transcribed using the iScript cDNA Synthesis Kit (Bio‐Rad, Hercules, CA, USA). The final mixture was incubated at 25 °C for 5 min, at 42 °C for 20 min, at 47 °C for 20 min, at 50 °C for 15 min, and at 85 °C for 5 min. Real‐Time PCR was performed on the 7500 Real‐Time PCR System using the SYBR Select Master Mix (Applied Biosystems, Foster City, CA, USA). Primers sequences are reported in Table S1. Amplification was performed in a final volume of 20 µL containing 2× Gene expression master Mix (Applied Biosystem), 2 µL of cDNA in a total volume of 20 µL. The reaction mixtures were all subjected to 2 min at 50 °C, 10 min at 95 °C followed by 40 PCR cycles at 95 °C for 15 s and 60 °C for 1 min for overall markers. The amount of transcripts was normalized to the endogenous reference genes β‐actin and HPRT and expressed as n‐fold mRNA levels relative to a calibrator using a comparative threshold cycle (Ct) value method (∆∆Ct). The RNA extracted from untreated cells was used as the calibrator.

### 2.6 Flow cytometry

Cells were collected by trypsinization for monolayer culture or by digestion in Collagenase type I (Merck Millipore) for 3D culture. To determine cell viability, cells were stained with 50 µm calcein‐AM and 2 mm ethidium homodimer‐1 (Invitrogen). For lysosomes quantification, cells were stained with 50 nm LysoTracker™ Green DND‐26 (Thermo Fisher Scientific). The TUNEL assay was performed with the In Situ Cell Death Detection Kit (Roche, Basel, Switzerland) according to the manufacturer's protocol. The cell suspensions were analyzed on the BD FACS CantoI (Beckmann Coulter, Brea, CA, USA).

### 2.7 Immunohistochemical analysis

Scaffolds were fixed in neutral buffered formalin, dehydrated by incubation in scaling ethanol solutions (30–100%), and embedded in paraffin as previously described [34]. Paraffin blocks were sliced with a rotating microtome (Leica Biosystems, Wetzlar, Germany) at 5 µm thickness, and sections were mounted onto Superfrost Plus microslides (Thermo Fisher Scientific, Waltman, MA, USA). Hematoxylin and eosin staining was performed to assess the scaffold architecture, cell morphology, and distribution. Immunostaining for anti‐HIF‐1α (1 : 500, Abcam) was performed using the Ventana Benchmark XT staining system (Ventana Medical Systems, Tucson, AZ, USA) with the Optiview DAB Detection Kit (Ventana Medical Systems).

### 2.8 Western blot

Proteins were isolated with a lysis buffer composed of 50 mm Tris/HCl (pH 8), 150 mm NaCl, 1% Triton X‐100, and 0.1% SDS, supplemented with the Halt Protease and Phosphatase Inhibitor Cocktail (Thermo Fisher Scientific). The protein content was quantified using the BCA protein assay kit (Thermo Fisher Scientific). For each sample, an equal amount of protein was loaded on Bolt™ 10% Bis‐Tris Plus Gels (Life Technologies) and transferred to polyvinylidene fluoride membranes through Trans‐Blot^®^ Turbo™ blotting system (Bio‐Rad). The membranes were blocked in 5% nonfat dry milk PBS with 0.1% Tween 20 (Sigma‐Aldrich) for 2 h at room temperature. Then, the membranes were incubated overnight with primary antibodies at 4 °C. The following antibodies were used: anti‐CASP3 (1 : 1000; Cell Signaling Technology) and anti‐vinculin (1 : 1000; Thermo Fisher Scientific). After two washes, the membranes were incubated for 1 h at room temperature with horseradish peroxidase‐conjugated secondary antibody followed by the visualization of the proteins with a chemidoc XRS system (Bio‐Rad).

### 2.9 Doxorubicin testing

Doxorubicin treatment was performed in monolayer cultures or in the 3D scaffolds at the following concentrations: 0.8, 1.6, and the human plasma peak concentration 4 μg·mL^−1^ [41, 42]. Doxorubicin hydrochloride solution was diluted in culture media. Cells were cultured for 24 h before exposure to the drug. Cell viability was assessed after 72 h of treatment (according to the terminal half‐life of doxorubicin) [43] by MTT assay directly in the scaffolds or in the culture wells. Briefly, controls and drug‐treated samples were incubated with 0.5 mg·mL^−1^ of MTT solution (Sigma‐Aldrich) in DMEM for 2 h at 37 °C. Cell viability was determined by reading the absorbance at 550 nm. Survival percentages were calculated as the average absorbance of cells at each doxo doses over the absorbance of untreated cells.

*In vivo* experiments were performed through orthotopically injections into the right mammary fat pad of 6‐week‐old female immunodeficient NU/NU nude mice (Crl:NU‐Foxn1nu) purchased from Charles River Laboratories. 2 × 10^6^ MDA‐MB‐231 and MCF‐7 cells were marked with Luciferase and suspended in 100 µL Matrigel (BD) before the injection. Mice were maintained under pathogen‐free conditions and on low‐fluorescence diet according to the guidelines set forth by the National Institutes of Health. Tumor growth was followed by *in vivo* bioluminescence imaging using the Xenogen IVIS 200 In Vivo Bioluminescence Imaging System (PerkinElmer, Waltham, MA, USA) every 2–3 days after cells injection. Tumor volume was assessed at each time point by caliper measurement. When the tumors reached an average volume of 70 mm^3^, animals were randomly assigned to either control or doxo group (5 mice per experimental group). Doxorubicin hydrochloride was dissolved in saline and administered daily by intraperitoneal injection at the doses of 0.2, 0.08, and 0.04 mg·kg^−1^ (dosages were selected according to the human plasma peak of doxorubicin from pharmacokinetic clinical data and converted to mice equivalent surface area) [44], while control animals were injected with the same volume of saline. After 3 days, the treatment was stopped and after 1 week animals were sacrificed (Fig. S1). The percentages of cell survival were calculated normalizing the average volume of treated tumors versus the average volume of untreated controls. Tumors were collected, fragmented into small pieces, and stored in TRIzol at −80 °C for RNA extraction or in lysis buffer at −80 °C for protein extraction.

For both *in vivo* and *in vitro* data, the IC_50_ values were calculated from the nonlinear regression of the dose–log response curves.

### 2.10 Statistics

For each experiment, at least three biologically independent replicates were performed. Data were presented as mean ± standard deviation (S.D.), or mean ± standard error of the mean (S.E.M.), as specified. *N* indicates the number of replicates. The differences between groups were assessed by two‐tailed Student's *t*‐test or Mann–Whitney test, as stated, and accepted as significant when *P* < 0.05.

### 2.11 Study approval

All animal procedures were reviewed and approved by the Institutional Animal Care and Use Committee (IACUC) of the Houston Methodist Research Institute (HMRI) protocol number AUP 0614‐0033.

## 3 Results

### 3.1 Efficacy of doxorubicin in 3D culture is analogous to *in vivo* response

**Fig. 1:**
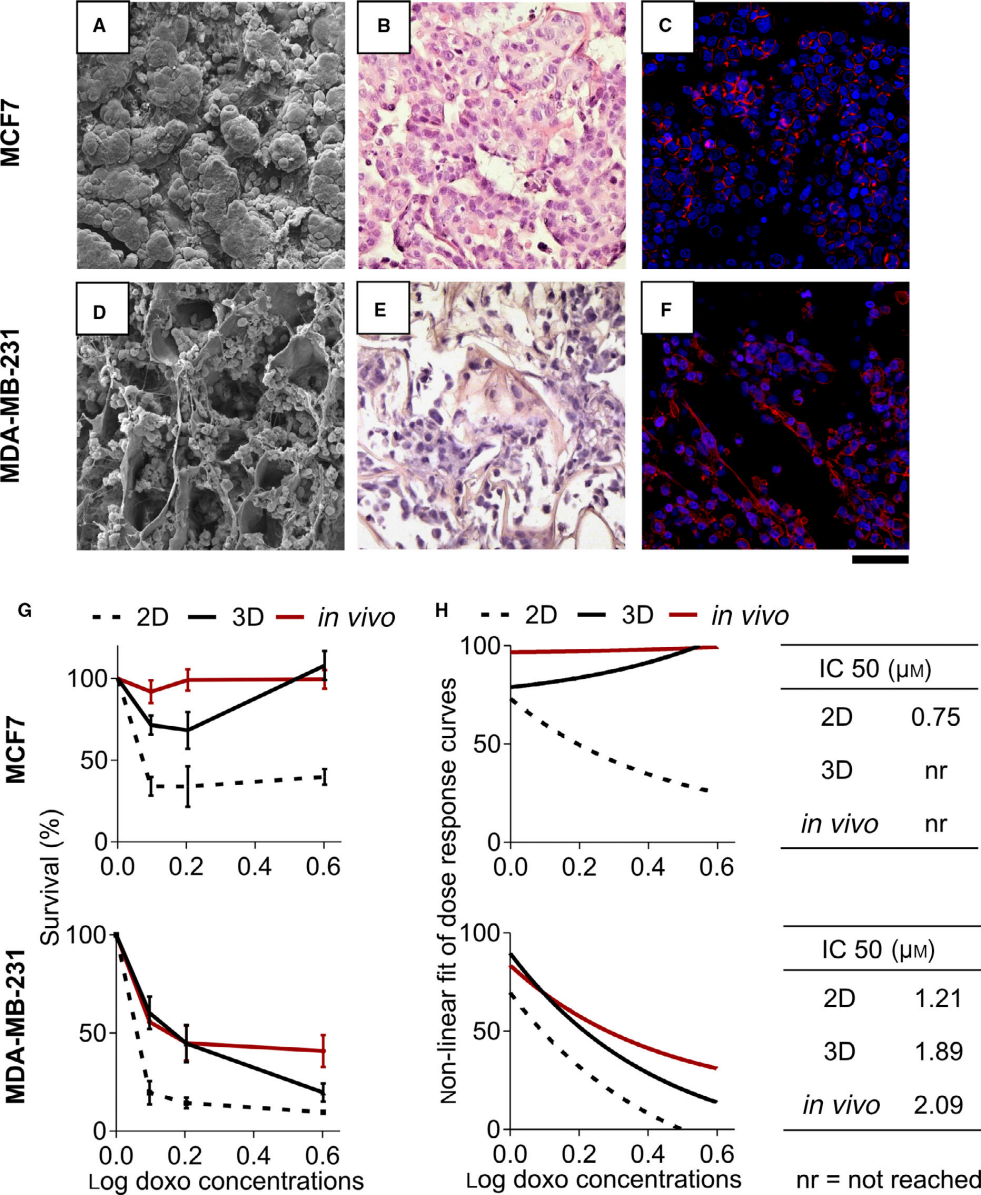
Characterization and drug sensitivity of breast cancer cells in 3D collagen scaffold. (A, D) SEM micrograph of collagen scaffolds showing the porous surface of the material cellularized with MCF‐7 (A) and MDA‐MB‐231 (B) (*n* = 3). (B, E) Hematoxylin‐and‐eosin–stained histological sections of MCF‐7 (B) and MDA‐MB‐231 (E) within the scaffolds at day 7 (*n* = 3). (C, F) Confocal microscopy images of MCF‐7 (C) and MDA‐MB‐231 (F) within the scaffold at day 7 (*n* = 3). Cells are stained with DAPI (blue) and phalloidin (red). Scale bars for all pictures: 100 µm. (G) Percentages of survival of MCF‐7 and MDA‐MB‐231 after 72 h of treatment with different concentrations of doxorubicin in monolayer culture (2D), within the scaffold (3D), or orthotopically implanted into a murine model (*in vivo*). Data represent mean ± S.D. (*n* = 3 for *in vitro* data, *n* = 6 for *in vivo* data). (H) Nonlinear fit of log–dose responses curves and IC_50_ calculation.

Breast cancer cells cultured in collagen scaffolds recreated a tissue‐like organization with distinct cell phenotypes, as previously observed [34]. MCF‐7 grew in discrete round clusters with a tightly cohesive structure and displayed an epithelial morphology (Fig. 1A–C). MDA‐MB‐231 grew homogeneously dispersed within the scaffold's pores showing a spindle mesenchymal morphology (Fig. 1D–F). We compared the efficacy of doxorubicin (doxo), one of the most used chemotherapy agent for the treatment of breast cancer patients [45], in 3D culture, standard monolayer, and *in vivo*. Both cell lines showed within the scaffold a decreased sensitivity to the drug compared with cells in monolayer, as shown by the higher rates of survival at all tested concentrations (Fig. 1G) and by the IC_50_ values (Fig. 1H). Efficacy in the scaffold was analogous to that demonstrated by cells growing *in vivo* in terms of survival rates, dose–response curves, and IC_50_ values. Conversely, cells cultured in monolayer were much more sensitive to the drug. Notably, MCF‐7 demonstrated to be relatively insensitive to doxorubicin treatment in 3D and *in vivo* conditions being completely resistant at the highest dose: Cells treated with 4 µg·mL^−1^ doxorubicin showed a survival percentage near to 100% and the IC_50_ concentration was not reached (Fig. 1H).

Decreased sensitivity was not caused by impaired drug penetration in inner scaffold areas: No significant differences were observed in the mean fluorescence intensity of doxorubicin between cells in core or edge regions of the scaffold, with the exception of the 0.8 µg·mL^−1^ dose in MDA‐MB‐231 (*P* = 0.032) (Fig. S1b).

### 3.2 Lineage‐specific signaling pathways are activated in doxorubicin‐treated cells within the scaffold

**Fig. 2:**
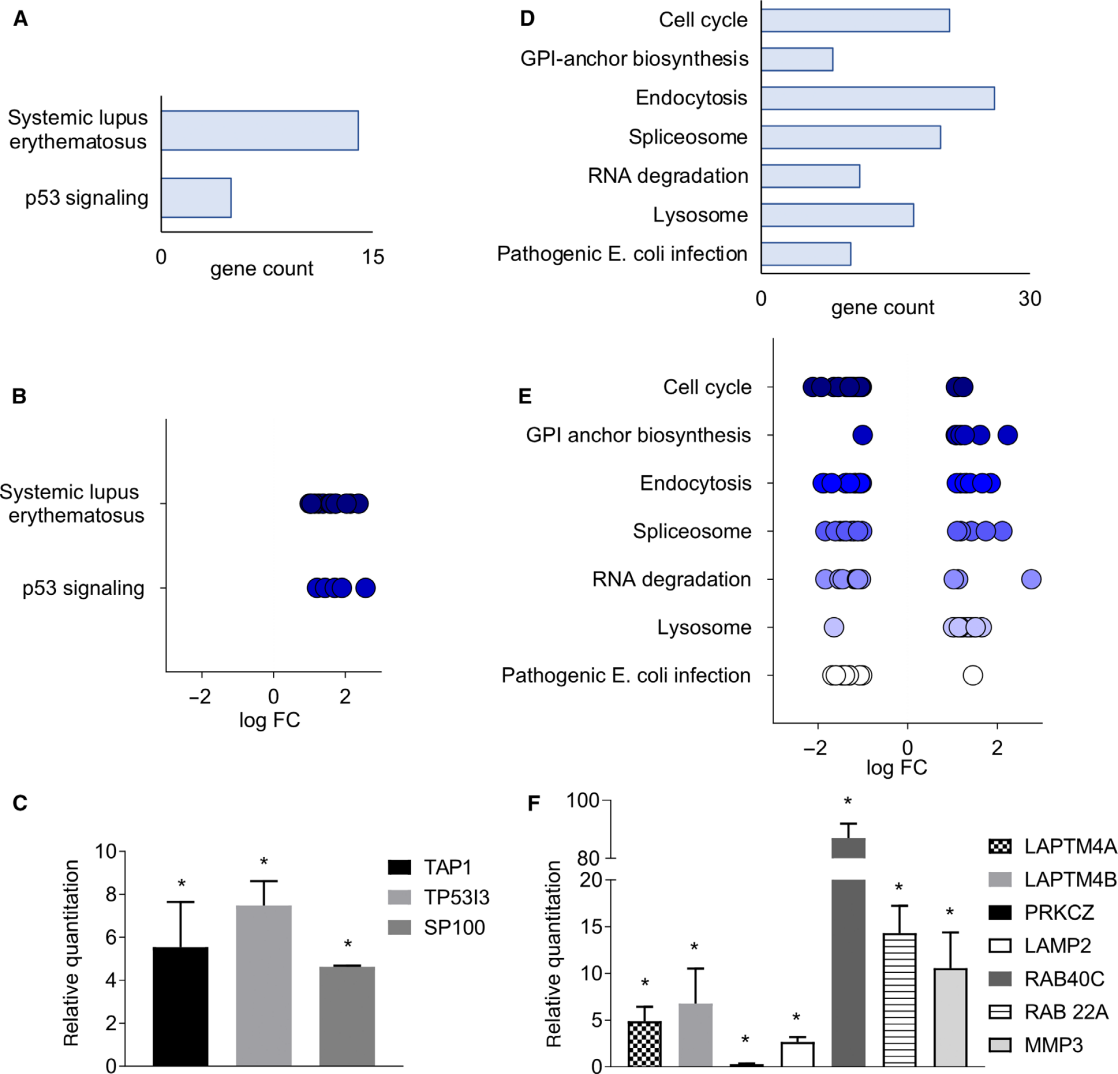
Transcriptomic data analysis of breast cancer cells treated with doxorubicin. (A, D) Gene count of significantly altered pathways identified in MCF‐7 (A) or MDA‐MB‐231 (D) treated with doxorubicin within 3D collagen scaffolds. (B, E) Log fold change of DEGs in each identified pathway for MCF‐7 (B) or MDA‐MB‐231 (E). (C, F) Relative expression levels from qPCR data of candidate DEGs belonging to the identified pathway for MCF‐7 (C) and MDA‐MB‐231 (F) treated with doxo. The values are relative to untreated control samples. Data represent mean ± S.D. (*n* = 3). *P < 0.05, two‐tailed Student's *t*‐test.

Diverse signaling pathways were found to be modulated in the two cell lines in response to doxorubicin administration. Transcriptome analysis demonstrated that MCF‐7 cultured in the scaffolds and treated with 4 µg·mL^−1^ doxo, at which cells were totally resistant, showed upregulation of the systemic lupus erythematosus and p53 signaling pathways (Fig. 2A,B). Between the most DEGs *TAP1*, *TP53I3*, *GADD45G*, *GADD45B*, and *S100P* were found. The expression levels of selected candidate DEGs were quantified by qPCR analysis (Table S1). All genes resulted significantly upregulated in treated cells compared with controls (*P* = 0.0461 for *TAP1*, *P* = 0.0075 for *TP53I3* and *P* = 0.0085 for *S100P*) (Fig. 2C). In MDA‐MB‐231, we observed the upregulation of the glycosylphosphatidylinositol (GPI)‐anchor biosynthesis and lysosome pathways and downregulation of pathways involving cell cycle, endocytosis, spliceosome, RNA degradation, and Pathogenic *Escherichia coli* infection (Fig. 2D,E). Between the most deregulated genes, *LAPTM4A*, *LAPTM4B*, *PRKCZ*, *LAMP2*, *RAB40C*, *RAB22A*, and *MMP3* were found. The qPCR analysis on selected DEGs (Table S2) confirmed that *LAPTM4A*, *LAPTM4B*, *LAMP2*, *RAB40C*, *RAB22A*, and *MMP3* were significantly upregulated in treated samples compared with controls, while *PRKCZ* was downregulated (*P* = 0.01119 for *LAPTM4A*, *P* = 0.01833 for *LAPTM4B*, *P* = 0.02232 for *LAMP2*, *P* = 0.01293 for *RAB40C*, *P* = 0.00779 for *RAB22A*, *P* = 0.03562 for *MMP3* and *P* = 0.00169 for *PRCKZ*) (Fig. 2F). The identified markers were not affected when cells were treated with doxorubicin in monolayer culture with the exception of *PRKCZ* that resulted downregulated in treated samples (*P* = 0.03844), as observed within the scaffolds (Fig. S1c).

### 3.3 Reactivation of caspase 3 and p53 signaling induction in drug‐resistant MCF‐7

**Fig. 3:**
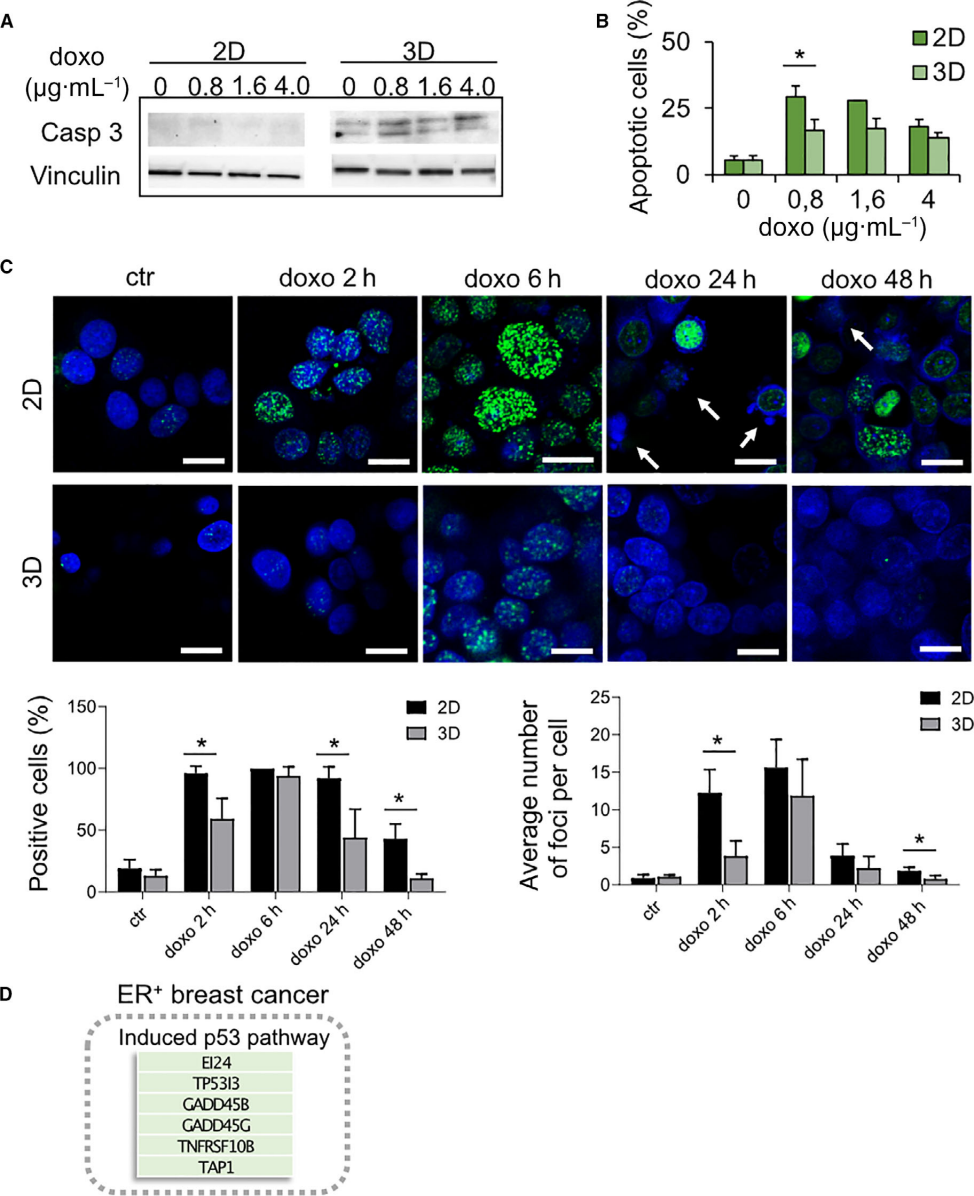
Mechanism of drug resistance in MCF‐7 cultured within the scaffold. (A) Western blot for caspase 3 in MCF‐7 untreated or treated with different concentrations of doxorubicin for 72 h in monolayer culture (2D) or within the scaffold (3D). (B) Percentages of apoptotic MCF‐7 after 72 h of treatment with different concentrations of doxorubicin in 2D or 3D cultures. Data represent mean ± S.D. (*n* = 3). *P < 0.05, two‐tailed Student's *t*‐test. (C) Immunofluorescence staining of γH2AX in MCF‐7 cells untreated (ctr) or treated with doxorubicin for 2, 6, 24, and 48 h in 2D or 3D cultures; quantification of the percentages of γH2AX‐positive cells and of the average number of γH2AX foci per cell. Data represent mean ± S.E.M (*n* = 5). *P < 0.05, two‐tailed Student's *t*‐test. Scale bars for all pictures: 20 µm. Arrows indicate apoptotic cells. (D) Schematic representation of doxorubicin effects in MCF‐7 cell line cultured within the scaffold. The most significantly altered pathway implicated in drug resistance with the list of relative DEGs are reported in the box. Green is indicative of upregulation.

In MCF‐7, the selection of a doxorubicin‐resistant subpopulation from parental cells was observed, as demonstrated by the detection of caspase 3 protein in cells cultured in the scaffold (Fig. 3A). MCF‐7 are normally known to express a truncated isoform of caspase 3, while the drug‐resistant sublines express the full‐length transcript [46]. Cell selection process was partially independent from drug exposure as cells expressing full‐length caspase 3 were present also in control samples, although to a lower extent. Despite the expression of caspase 3, cells within the scaffold showed lower levels of apoptotic cell death after drug exposure, compared with cells in monolayer (*P* = 0.01434 for the 0.8 µg·mL^−1^ dose) (Fig. 3B). We, thus, investigated the activation of DNA damage response. Doxorubicin treatment induced an increase in the accumulation of γH2AX foci, which is indicative of DNA double‐strand breaks, in both monolayer and 3D‐cultured cells. However, DNA damage response activation and apoptosis detection were significantly lower in cells treated within the scaffold compared with monolayer, with faster resolution of γH2AX foci (Fig. 3C). The number of cells positive for γH2AX and the average number of foci were significantly lower within the scaffold, in particular at later time points (*P* = 0.0032 at 2 h, *P* = 0.0048 at 24 h, and *P* = 0.0098 at 48 h after treatment for the number of positive cells; *P* = 0.0032 at 2 h, and *P* = 0.0151 at 48 h after treatment for the average number of foci) (Fig. 3C). Conversely, in monolayer resolution of γH2AX foci of DNA, breaks occurred at a later time point (48 h), and apoptotic cells were detected after 24 h (arrowed) (Fig. 3C). Caspase 3 expression and induction of p53 signaling (Fig. 3D) in cells treated within the scaffold did not result in significant DNA damage response activation and apoptosis.

### 3.4 MDA‐MB‐231 show reduced doxorubicin uptake and lysosomal confinement of the drug

**Fig. 4:**
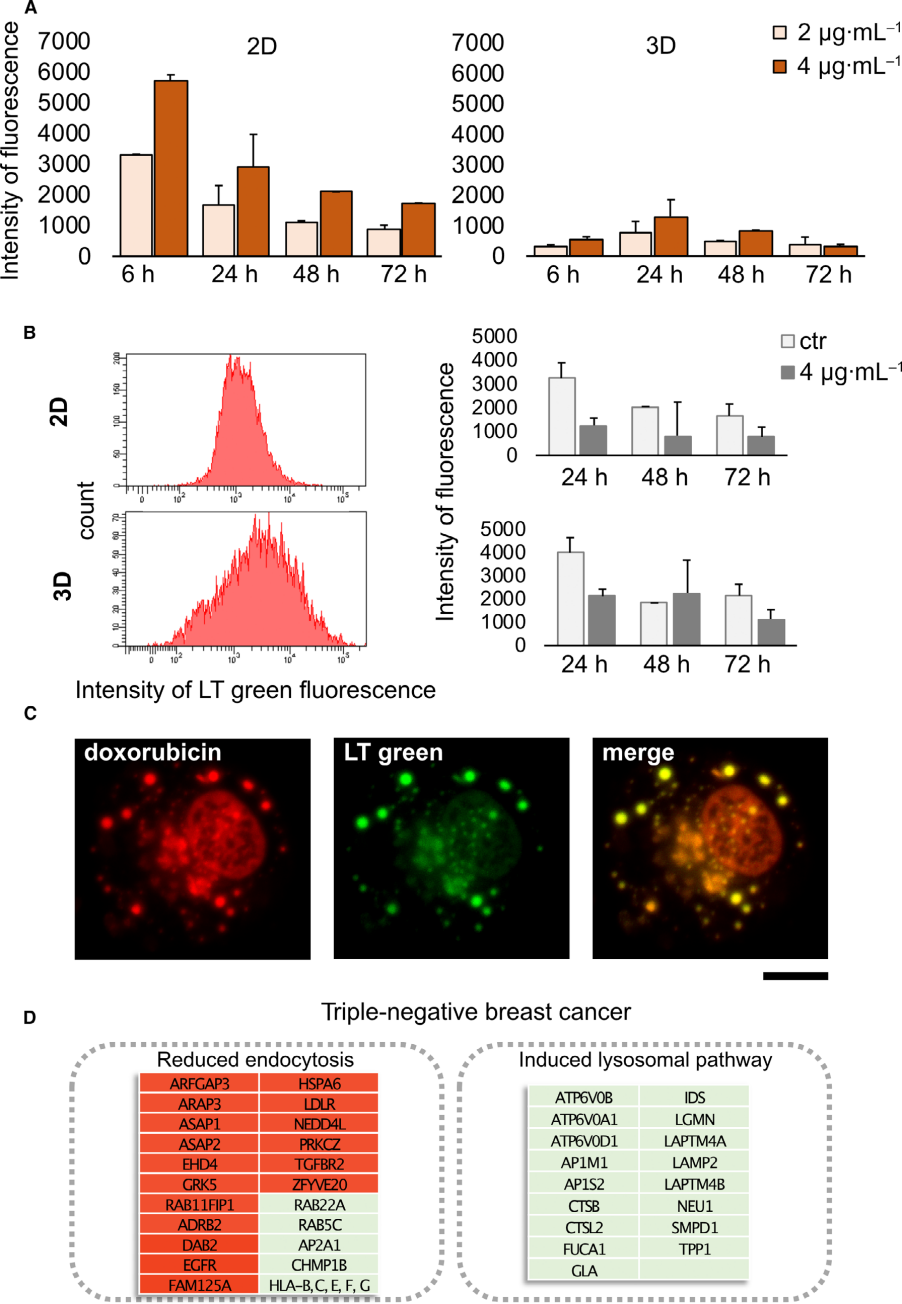
Mechanism of drug resistance in MDA‐MB‐231 cultured within the scaffold. (A) Doxorubicin median fluorescence intensity detected by flow cytometry in MDA‐MB‐231 after 6, 24, 48, and 72 h of treatment with different doxo concentrations in monolayer culture (2D) or within the scaffold (3D). Data represent mean ± S.D. (*n* = 3). (B) Histogram plot of MDA‐MB‐231 stained with lysotracker (LT) green in 2D or 3D cultures and median fluorescence intensity of LT green in control cells or cells treated with 4 µg·mL^−1^ doxorubicin after 24, 48, and 72 h. Data represent mean ± S.D. (*n* = 3). (C) Confocal microscopy images of MDA‐MB‐231 treated with doxorubicin within the scaffold. Red is doxorubicin autofluorescence and green is LT green signal. Scale bar is 10 µm (D) Schematic representation of doxorubicin effects in MDA‐MB‐231 cell line cultured within the scaffold. The most significantly altered pathways implicated in drug resistance with the list of relative DEGs are reported in the box. Green is indicative of upregulation. Red is indicative of downregulation.

MDA‐MB‐231 treated with doxorubicin in the scaffold showed a reduced intracellular accumulation of the drug compared with cells in monolayer, as demonstrated by lower doxorubicin fluorescence signal detected by flow cytometry at different time points after administration (Fig. 4A). These data were confirmed by fluorescence microscopy analysis of treated cells recovered from monolayer culture or from the scaffold (Fig. S2A). In monolayer, doxorubicin signal decreased over time, while it was relatively constant for cells in 3D. Interestingly, we observed the presence of a cell side population characterized by a low‐fluorescence intensity both in the doxorubicin and in the calcein‐AM fluorescence channels (Fig. S2B). Calcein is known to be extruded by the multidrug transporter MDR‐1 before the intracellular conversion to its fluorescent‐free isoform and provides an efficient experimental method to determine the activity of MDR‐1 in cancer cells [47]. The percentage of cells showing this phenotype was significantly lower in monolayer culture (*P* = 0.045 and *P* = 0.039 at 0 and 4 µg·mL^−1^ dose, respectively). Moreover, we found that the lysosomal content of cells cultured within the scaffold was significantly higher compared with cells in monolayer (Fig. 4B and Fig. S2C). Confocal microscopy analysis demonstrated the colocalization of the fluorescence signal of doxorubicin with labeled lysosomes (Fig. 4C). These mechanisms were partially independent from drug exposure, as all observed phenotypes were detected also in untreated samples, although to a lower extent. Taken together, these data are consistent with the reduced endocytosis and the induction of lysosomal pathway observed by transcriptome analysis in cells treated within the scaffold (Fig. 4D).

### 3.5 Hypoxia is involved in doxorubicin resistance

**Fig. 5:**
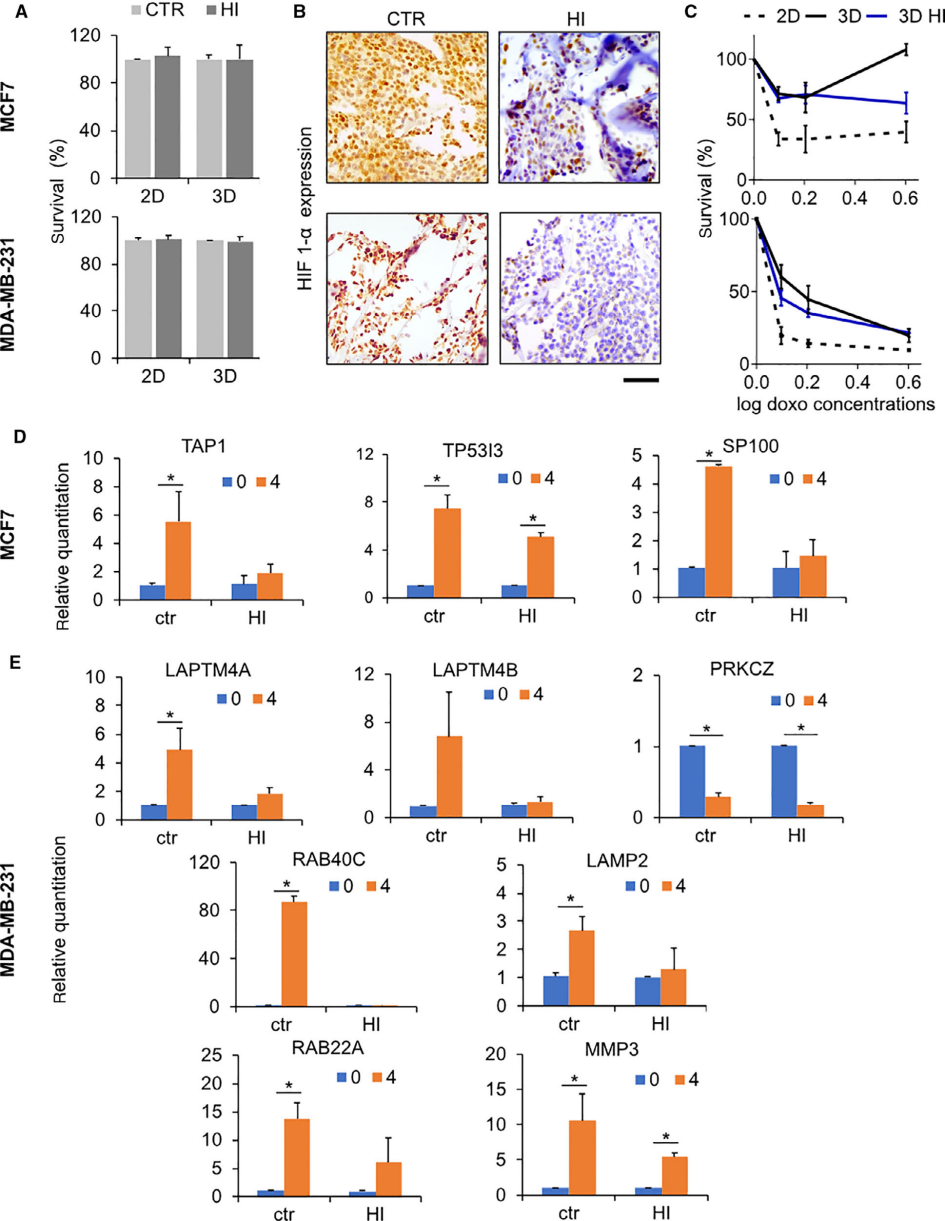
Role of hypoxia in 3D‐induced drug resistance. (A) Survival percentages (day 7) of MCF‐7 and MDA‐MB‐231 cultured in monolayer (2D) of within the scaffold (3D) in the absence (CTR) or presence of a hypoxia inhibitor (HI). Data represent mean ± S.D. (*n* = 5). (B) HIF‐1α expression in histological sections of MCF‐7 and MDA‐MB‐231 cultured within the scaffold in control conditions (CTR) or in the presence of an hypoxia inhibitor (HI). Scale bars: 50 µm. (C) Percentage of survival of MCF‐7 and MDA‐MB‐231 after 72 h of treatment with different concentrations of doxorubicin in monolayer culture (2D), within the scaffold (3D) and in the presence (3D HI) of a hypoxia inhibitor. Data represent mean ± S.D. (*n* = 3). (D) Relative expression levels from qPCR data of candidate DEGs in MCF‐7 untreated or treated with 4 µg·mL^−1^ doxorubicin under control conditions (ctr) or in the presence of hypoxia inhibition (HI). Data represent mean ± S.D. (*n* = 3). *P < 0.05, two‐tailed Student's *t*‐test. (E) Relative expression levels from qPCR data of candidate DEGs in MDA‐MB‐231 untreated or treated with 4 µg·mL^−1^ doxorubicin under control conditions (ctr) or in the presence of hypoxia inhibition (HI). Data represent mean ± S.D. (*n* = 3). *P < 0.05, two‐tailed Student's *t*‐test.

We previously observed that our 3D model allows for the creation of a hypoxic core environment that guides multiple phenotypic changes in breast cancer cells [34]. To address the correlation between hypoxia and the emergence of chemotherapy resistance in 3D‐cultured cells, we performed treatment in the presence of Resveratrol, a hypoxia inhibitor that reduces hypoxia‐mediated HIF‐1α accumulation [48]. A preincubation of 24 h with 50 µm Resveratrol was performed prior to drug administration. While Resveratrol did not affect cancer cell proliferation either in the scaffold or in monolayer cultures (Fig. 5A), it markedly decreased HIF‐1α expression in both cell lines (Fig. 5B). Hypoxia inhibition was found to re‐sensitize 3D‐cultured cells to doxorubicin, as proved by the marked decrease in survival percentages demonstrated by MCF‐7 at the highest drug dose and by MDA‐MB‐231 at the doses of 0.8 and 1.6 µg·mL^−1^ (Fig. 5C). Sensitivity was not completely restored as that observed for monolayer cultured cells, suggesting that other 3D‐related phenotypes, such as the slower rate of proliferation, might contribute to the induction of resistance.

Moreover, the hypoxia inhibition reduced the expression level of all the biomarkers involved in resistance mechanisms. After doxorubicin treatment under hypoxia, *TAP1* and *S100P* were not significantly overexpressed in MCF‐7 cell line, in contrast to control conditions (Fig. 5D). Conversely, expression of *TP53I3* was significantly enhanced even when hypoxia was inhibited. In MDA‐MB‐231, the differences in expression of *LAPTM4A*, *LAPTM4B*, *LAMP2*, *RAB40C*, and *RAB22A* between control and treated samples resulted not significant under hypoxia inhibition, in contrast to what observed for samples treated under control conditions (Fig. 5E). On the contrary, a significant downregulation of *PRKCZ* and overexpression of *MMP3* was observed even when hypoxia was inhibited. Interestingly, when cancer cells are pretreated with hypoxia inhibitors, there is a significant downregulation of most of the markers in response to doxorubicin treatment in cells. This result suggests a direct correlation between hypoxia and drug exposure in the acquired resistance by MDA‐MB‐231.

### 3.6 The identified biomarkers are involved in doxorubicin response *in vivo* and in breast cancer patients

**Fig. 6:**
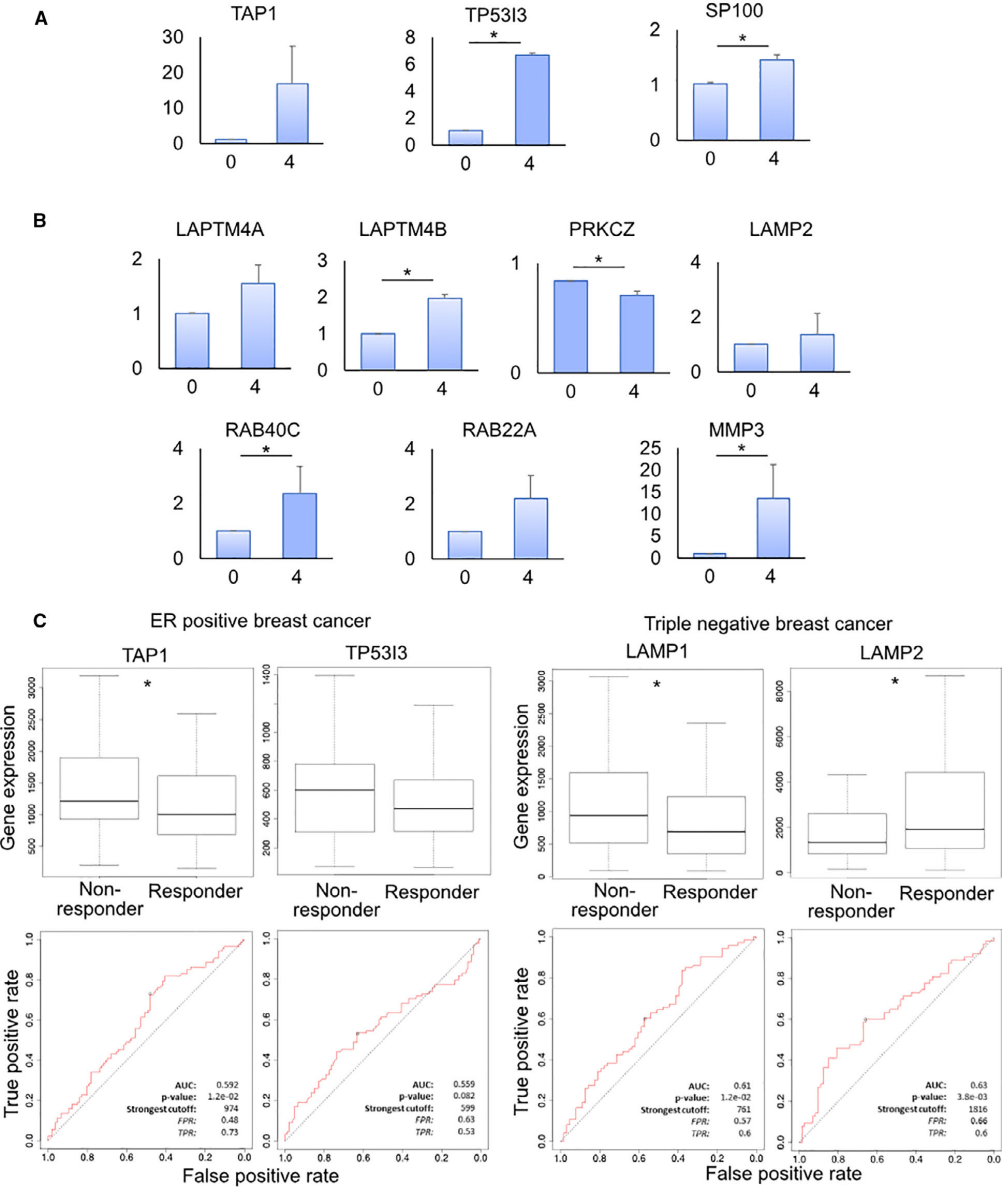
Expression levels of the identified biomarkers *in vivo* and in breast cancer patients. (A) Relative expression levels from qPCR data of candidate DEGs in MCF‐7 untreated or treated with 4 µg·mL^−1^ doxorubicin in an orthotopic murine model. Data represent mean ± S.D. (*n* = 3). *P < 0.05, two‐tailed Student's *t*‐test. (B) Relative expression levels from qPCR data of candidate DEGs in MDA‐MB‐231 untreated or treated with 4 µg·mL^−1^ doxorubicin in an orthotopic murine model. Data represent mean ± S.D. (*n* = 3). *P < 0.05, two‐tailed Student's *t*‐test. (C) Expression levels of *TAP1* and *TP53I3* in ER‐positive breast cancer patients and *LAMP1* and *LAMP2* in triple‐negative breast cancer patients in relation to response to anthracycline treatment. Patients were classified as responder or nonresponder according to the 5‐year relapse‐free survival. ROC curves of *TAP1* and *TP53I3* as predictor of response to anthracycline treatment in ER‐positive breast cancer patients, and *LAMP1* and *LAMP2* as predictor of response to anthracycline treatment in triple‐negative breast cancer patients. *P < 0.05, two‐tailed Student's *t*‐test, Mann–Whitney test.

In order to understand their translational significance, we analyzed the expression of all biomarkers involved in resistance in orthotopic tumors generated in murine models and in a cohort of breast cancer patients from a public dataset. In *in vivo* samples, ER+ tumors treated with doxorubicin displayed significant upregulation of *TP53I3* and *S100P*, compared with untreated samples. Also *TAP1* was upregulated by treatment although the data were not statistically significant (Fig. 6A). In MDA‐MB‐231, we found a significant upregulation of *LAPTM4B*, *RAB40C*, *MMP3*, and downregulation of *PRKCZ* after tumor treatment with doxorubicin. Conversely, expression of *LAPTM4A*, *LAMP2* and *RAB22A* resulted not significantly affected (Fig. 6B).

For the expression analysis in breast cancer patients, we used the online transcriptome‐level validation tool for predictive biomarkers ROC Plotter that integrates 3104 breast cancer patients with treatment and response data [49]. We investigated the correlation between expression of the biomarkers identified in our screening and response to anthracycline regimens by means of relapse‐free survival at 5 years. Each biomarker was investigated in patients with the matching molecular subgroups. In ER^+^ breast tumor, we found that patients who did not respond to therapy showed a higher expression of *TAP1* and *TP53I3* compared with responders. High expression of *TAP1* and *TP53I3* was associated with shorter relapse‐free survival after treatment (Fig. 6C). In triple‐negative breast cancer, we found that patients who did not respond to therapy showed a higher expression of *LAMP1*, but lower expression of *LAMP2* compared with responders. High expression of *LAMP1* was associated with shorter relapse‐free survival after treatment (Fig. 6C). All other identified markers did not show a significant deregulation in this dataset of patients (data not shown).

## 4 Discussion

Engineered 3D models are generating increasing knowledge on drug sensitivity and on mechanisms of resistance acquisition in cancer cells, while offering high‐throughput analyses and cost‐efficient screenings [50, 51, 52, 53, 54, 55]. These innovative experimental models have represented a groundbreaking innovation for the clinical translation of anticancer agents. Here, we used a 3D technology based on biomimetic collagen scaffolds, enabling the modeling of the tumor hypoxic niche, to identify and describe mechanisms and drivers of chemotherapy resistance in breast cancer. Firstly, we demonstrated that *in vitro* results from our 3D model were comparable to those obtained using murine tumor xenografts. The activity of doxorubicin, one of most used chemotherapy agent for the treatment of breast cancer patients, tested in 3D was predictable of *in vivo* response. Conversely, efficacy was significantly overestimated when tested in monolayer culture. *In vivo* models remain the gold standard for preclinical drug development, despite showing the important drawbacks of time‐consuming, high cost, and availability depending on the tumor type [56]. The development of more reliable *in vitro* systems is reducing the amount of animals required for pharmacological trials, allowing to generate data with comparable translational value. In particular, an interesting observation was that the ER^+^ luminal A cell line demonstrated poor responsiveness to doxorubicin within our cancer model and in tumor xenografts. This is consistent with emerging clinical evidence that indicates the potential lack of benefit from anthracycline chemotherapy in patients with ER^+^ luminal A breast tumors [57, 58, 59, 60].

Through our 3D model, we next described the mechanisms of resistance which were specifically activated in the two molecular subgroups of breast cancer. In ER^+^ positive cells, the selection of a drug‐resistant subpopulation was observed. The presence of inherently resistant subclones in parental MCF‐7 cells, characterized by the expression of full‐length *CASP3*, has already been demonstrated [46]. Here, we showed that culturing in our biomimetic model results in the selection and propagation of this resistant subclone. This subpopulation shows overexpression of *TP53I3* and *TAP1* correlated to multidrug resistance in human cancers [61, 62] and with the presence of hypoxic conditions [63]. *TP53I3* has been found to be involved in mitotic progression regulation in non–small‐cell lung cancer [62], while *TAP1* is a member of the superfamily of ATP‐binding cassette (ABC) transporters [61]. In particular, TAP complex possesses characteristics of a xenobiotic transporter and the TAP dimer contributes to the atypical MDR phenotype of human cancer cells, mediating the translocation of hydrophobic antitumor agents into the endoplasmic reticulum lumen [61]. These cells displayed also reduced DNA damage response, despite expression of caspase 3, indicating a potential increased ability of DNA repair. This was further suggested by the enhanced expression of the *GADD45* family, members of the p53 signaling pathway, and mediators of demethylation and DNA excision repair [64].

Triple negative cells were able to reduce the intracellular drug accumulation through different processes: the downregulation of endocytic pathway components and the selection of a side subpopulation displaying the ability of extruding calcein and doxorubicin. Side population cells have been identified in several human cancers and are defined as cells capable of extruding dyes, such as Hoechst 33342, through the ABC transporters [47, 65, 66]. These cells were found, not only to possess increased drug resistance but also to display stem‐like properties [67]. Culturing within our 3D environment results in a significant selection of side population cells offering the possibility to further understand their functional and molecular characteristics. In addition to reduced intracellular drug accumulation, we found these cells to activate the lysosomal pathway and to accumulate doxorubicin inside lysosomes. This mechanism of resistance, identified in cisplatin‐treated cancer cells [68], has not yet been described for anthracyclines and hold an interesting potential. It has been demonstrated that mammalian target of rapamycin complex 1 (mTORC1), a downstream effector of oncogenic pathways, directly regulates the lysosomal biogenesis [69]. Several compounds able to suppress mTORC1 functions, as everolimus and temsirolimus, have been developed and are currently in clinical practice [69]. Combinatorial regimens, by counteracting the development of resistance, can be more effective than single therapy and should be considered as the best treatment option for many cancer patients [29] in order to prevent the increasing prevalence of drug resistance [70]. Here, we provide preliminary data to support the clinical rationale to explore the combination of doxorubicin and mTOR inhibitors for the treatment of triple‐negative breast cancer patients. However, further analyses are needed to support this approach.

In both cell lines, the resistant subpopulations emerged independently from doxorubicin exposure denoting intrinsic mechanisms, while treatment enhanced the observed phenotypes. Indeed, we demonstrated that the pretreatment of cancer cells with a hypoxia inhibitor hamper the upregulation of the identified markers related to drug resistance in response to doxorubicin exposure, suggesting a direct correlation between hypoxia and drug treatment. Hypoxia showed a central role in promoting resistance acquisition, as the blocking of HIF‐1α partially restored drug sensitivity in both breast cancer subtypes and decreased the molecular alterations induced by treatment. The role of hypoxia in cancer drug resistance is well documented. It has been demonstrated that hypoxia can confer resistance by regulating a number of signaling pathways as apoptosis, autophagy, DNA damage, mitochondrial activity, p53, and drug efflux [71]. In breast cancer, it has been recently demonstrated that resistance is connected to an increased plasticity of cells mediated by hypoxia [72]. Therefore, the possibility to model this process when screening anticancer agents demonstrates a crucial value and will help gaining new insights into mechanisms and molecular drivers of drug resistance [73, 74, 75].

Finally, profiling of treated cancer cells within the scaffold led to the identification of candidate predictive biomarkers. Several evidences indicates that engineered 3D models can be useful approaches to study and identify drug resistance mechanisms to anticancer agents [76, 77, 78, 79, 80]. Here, we demonstrate that the molecular changes identified through our biomimetic model are (a) predictive of *in vivo* molecular alterations on tumor xenografts and (b) demonstrate clinical predictive potential. Indeed, some of the biomarkers identified in our screening showed a significant value in predicting the 5‐year relapse rate of patients with breast cancer treated with anthracyclines regimens. Although our analysis shows some limitations, as the lack of standardization of patient characteristics in public datasets, it provides a proof‐of‐concept of the clinical value of these biomarkers. Therefore, further validation in independent cohorts of patients, ideally considering a neoadjuvant setting, is warranted.

## 5 Conclusion

These findings suggest that our model might support *in vitro* trials for the translation of targeted therapies and anticancer compounds as it provides (a) more relevant data on efficacy and (b) enhanced understanding of resistance acquisition, one the major causes of chemotherapy failure in cancer patients [1]. Our cancer model recreates the emergence of resistance fostered by a hypoxic niche and allows for the investigation of potentially unexplored mechanisms involved in therapy response. This approach may offer therapeutic targets for the design of combinatorial therapies and introduce new predictive biomarkers for precision medicine.

## Conflict of interest

The authors declare no conflict of interest.

## Author contributions

CL, TI, and LM designed the study. CL, ADV, CS, GM, CC, AB, ADL, FLM, FF, MT, and ET acquired and analyzed the data. CL, DA, LM, and TI conceived all the experiments and interpreted the results. CL, LM, and TI drafted the manuscript. All authors read and approved the final manuscript.

### Peer Review

The peer review history for this article is available at https://publons.com/publon/10.1002/1878‐0261.13037.

## Supporting information


**Fig. S1.** Schedule of doxorubicin administration in orthotopic murine models, doxorubicin localization in the 3D model, and DEGs expression in monolayer cells. (a) Schematic representation of the schedule of doxorubicin administration in orthotopic murine breast cancer models, generated by the xenotransplantation of MCF‐7 and MDA‐MB‐231. (b) Median fluorescence intensity of doxorubicin detected by immunofluorescence in MCF‐7 and MDA‐MB‐231 within core or edge regions of the scaffold after 72‐h treatment. Data represent mean ± S.D. (*n* = 20) *P < 0.05, two‐tailed Student's t‐test. (c) Relative expression levels from qPCR data of candidate DEGs belonging to the identified pathway for MCF‐7 and MDA‐MB‐231 treated with doxo in monolayer cultures. The values are relative to untreated control samples. Data represent mean ± S.D. (*n* = 3). *P < 0.05, two‐tailed Student's t‐test.

**Fig. S2.** Lysosomal‐mediated doxorubicin resistance in MDA‐MB‐231. (a) Representative images and median fluorescence intensity of doxorubicin detected by immunofluorescence in MDA‐MB‐231 cultured in monolayer (2D) or within the scaffolds (3D) after 72 h treatment with different doses. Scale bar is 20 µm. (b) Flow cytometry scatter plot of 2D‐ and 3D‐cultured MDA‐MB‐231 untreated or treated with 4 µg/ml doxo: Samples were double stained with Calcein AM and Ethidium Bromide. SP indicate a side population negative for both signals. On the right, percentages of doxorubicin‐ and calcein‐negative cells (side population) in 2D or 3D‐cultured MDA‐MB‐231 untreated or treated with doxo. Data represent mean ± S.D. (*n* = 3). *P < 0.05, two‐tailed Student's t‐test. (c) Inverted microscopy images of MDA‐MB‐231 treated with doxorubicin in monolayer culture (2D) or within the scaffold (3D). Red is doxorubicin autofluorescence and green is LT green signal. Scale bar is 20 µm.

**Table S1.** List of DEGs found in MCF‐7.
**Table S2.** List of DEGs found in MDA‐MB‐231.

## Data Availability

The datasets generated and/or analyzed during the current study are available from the corresponding author on reasonable request.
