# Supplementary figures and images for: Lineage‐specific mechanisms and drivers of breast cancer chemoresistance revealed by 3D biomimetic culture
Source: Mol Oncol. 2021 Jul 10;16(4):921–39. doi: 10.1002/1878-0261.13037 (PMC8847989; doi:10.1002/1878-0261.13037)

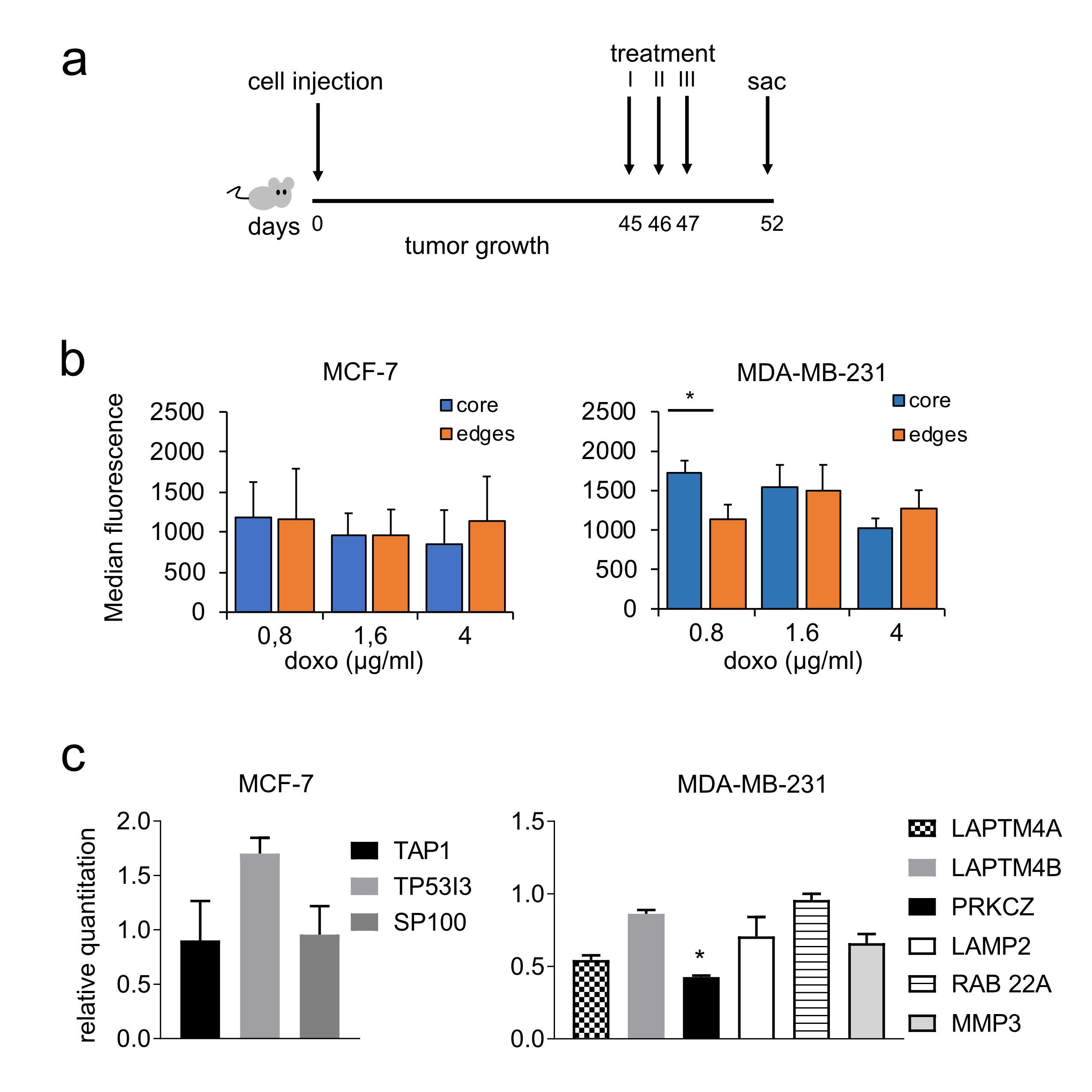

Supplement: Supplementary file 1 — Fig. S1. Schedule of doxorubicin administration in orthotopic murine models, doxorubicin localization in the 3D model, and DEGs expression in monolayer cells. (a) Schematic representation of the schedule of doxorubicin administration in orthotopic murine breast cancer models, generated by the xenotransplantation of MCF‐7 and MDA‐MB‐231. (b) Median fluorescence intensity of doxorubicin detected by immunofluorescence in MCF‐7 and MDA‐MB‐231 within core or edge regions of the scaffold after 72‐h treatment. Data represent mean ± S.D. (n = 20) *P < 0.05, two‐tailed Student's t‐test. (c) Relative expression levels from qPCR data of candidate DEGs belonging to the identified pathway for MCF‐7 and MDA‐MB‐231 treated with doxo in monolayer cultures. The values are relative to untreated control samples. Data represent mean ± S.D. (n = 3). *P < 0.05, two‐tailed Student's t‐test. [file MOL2-16-921-s001.tiff]

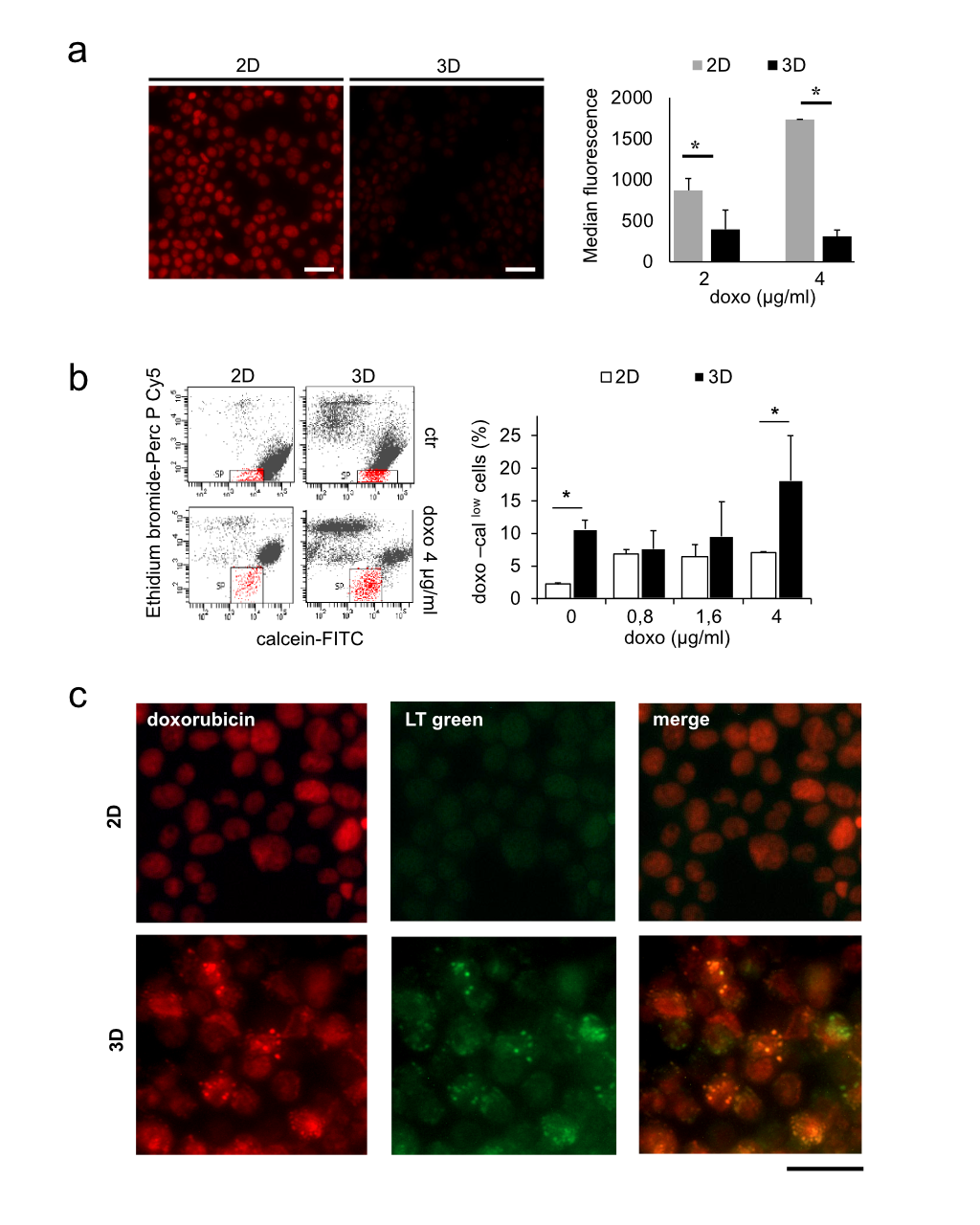

Supplement: Supplementary file 2 — Fig. S2. Lysosomal‐mediated doxorubicin resistance in MDA‐MB‐231. (a) Representative images and median fluorescence intensity of doxorubicin detected by immunofluorescence in MDA‐MB‐231 cultured in monolayer (2D) or within the scaffolds (3D) after 72 h treatment with different doses. Scale bar is 20 µm. (b) Flow cytometry scatter plot of 2D‐ and 3D‐cultured MDA‐MB‐231 untreated or treated with 4 µg/ml doxo: Samples were double stained with Calcein AM and Ethidium Bromide. SP indicate a side population negative for both signals. On the right, percentages of doxorubicin‐ and calcein‐negative cells (side population) in 2D or 3D‐cultured MDA‐MB‐231 untreated or treated with doxo. Data represent mean ± S.D. (n = 3). *P < 0.05, two‐tailed Student's t‐test. (c) Inverted microscopy images of MDA‐MB‐231 treated with doxorubicin in monolayer culture (2D) or within the scaffold (3D). Red is doxorubicin autofluorescence and green is LT green signal. Scale bar is 20 µm. [file MOL2-16-921-s003.tiff]
