# Supplementary material for: Lineage‐specific mechanisms and drivers of breast cancer chemoresistance revealed by 3D biomimetic culture
Source: Mol Oncol. 2021 Jul 10;16(4):921–39. doi: 10.1002/1878-0261.13037 (PMC8847989; doi:10.1002/1878-0261.13037)
Supplement: Supplementary file 3 — Table S1. List of DEGs found in MCF‐7. Table S2. List of DEGs found in MDA‐MB‐231. [file MOL2-16-921-s002.docx]

**Table S1.** List of DEGs found in MCF-7.

| **Gene** | **Pathway** | **Name** |
| --- | --- | --- |
| TAP1 | p53 signaling | Antigen Peptide Transporter 1 |
| TP53I3 | p53 signaling | Tumor Protein P53 Inducible Protein 3 |
| S100P | no pathway | S100 calcium-binding protein P |

**Table S2** List of DEGs found in MDA-MB-231

| **Gene** | **Pathway** | **Name** |
| --- | --- | --- |
| LAPTM4A | Lysosomes | lysosomal protein transmembrane 4 alpha |
| LAPTM4B | Lysosomes | lysosomal protein transmembrane 4 beta |
| PRKCZ | Endocytosis | Protein Kinase C Zeta |
| LAMP2 | Lysosomes | lysosomal-associated membrane protein 2 |
| RAB40C | no pathway | Ras-related protein Rab-40C |
| RAB22A | Endocytosis | Regulation of endosomal compartments |
| MMP-3 | no pathway | Matrix metalloproteinase 3 |
